# Maximal inspiratory diaphragmatic ultrasound predicts postoperative pulmonary complications after upper abdominal surgery

**DOI:** 10.1186/s13613-025-01531-2

**Published:** 2025-08-18

**Authors:** Ting Yan, Qing Yu, Chun-Qing Li, Zhen-Zhen Xu, Jia-Hui Ma, Min Xie, Sai-Nan Zhu, Dong-Xin Wang, Shuang-Ling Li

**Affiliations:** 1 Department of Critical Care Medicine, Peking University First Hospital, Beijing, 100034 China; 2 Department of Respiratory and Critical Care Medicine, Peking University First Hospital, Beijing, China; 3 Department of Anesthesiology, Peking University First Hospital, Beijing, China; 4 Department of Medical Statistics, Peking University First Hospital, Beijing, China

**Keywords:** Diaphragmatic ultrasonography, Postoperative pulmonary complications, ARISCAT, Perioperative care, Post-RDS-DE, Upper abdominal surgery

## Abstract

**Background:**

Postoperative pulmonary complications (PPCs) after major upper abdominal surgery are an important cause of morbidity and mortality. However, existing preoperative risk models inadequately address perioperative factors. Although diaphragmatic ultrasonography offers real-time assessment of respiratory muscle function, its predictive utility for PPCs remains underexplored. This study aimed to evaluate the predictive value of diaphragmatic ultrasound parameters for PPCs and to identify the optimal index among them.

**Methods:**

This prospective observational cohort study included patients aged ≥ 50 years who underwent elective upper abdominal surgery under general anesthesia. Right-sided diaphragmatic ultrasound evaluations were performed on preoperative day 1 (PreD1) and on postoperative day 1 (POD1), and measured diaphragm thickening fraction (DTF) and diaphragmatic excursion (DE) during quiet, deep, and sniff breathing. Patients were followed up for 14 days after surgery to assess the incidence of PPCs. Receiver operating characteristic (ROC) analysis and multivariate logistic regression were used to evaluate predictive performance and adjust for confounders.

**Results:**

Among the 223 patients enrolled, 37 (16.6%) developed PPCs. In the entire cohort, all parameters of diaphragmatic ultrasound showed significant postoperative reductions on POD1 compared to preoperative values *(P* < 0.001). ​A composite index (post-RDS-DE), calculated as the sum of right DEs during deep breathing and sniff breathing on POD1, demonstrated a moderate predictive ability for PPCs (AUC = 0.680, 95% CI: 0.587–0.773). At a cutoff value of post-RDS-DE < 3.55 cm, the negative predictive value reached 90.6%. ​​After multivariable adjustment, post-RDS-DE < 3.55 cm remained an independent predictor of PPCs (adjusted OR = 2.547, 95% CI: 1.067–6.080; *P* = 0.035).​​ Integration of diaphragmatic ultrasound index (post-RDS-DE < 3.55 cm) with the ARISCAT significantly improved predictive performance (AUC = 0.751 with integrated model vs. 0.643 with ARISCAT alone; DeLong’s *P* = 0.004).

**Conclusions:**

Postoperative maximal inspiratory diaphragmatic ultrasound measurements during deep and sniff breathing (quantified by a composite index, the post-RDS-DE) effectively predict PPCs following upper abdominal surgery. Integration of post-RDS-DE with preoperative ARISCAT markedly enhances predictive accuracy, suggesting diaphragmatic ultrasonography as a bedside tool for perioperative respiratory risk assessment.

**Supplementary Information:**

The online version contains supplementary material available at 10.1186/s13613-025-01531-2.

## Introduction

The incidence of postoperative pulmonary complications (PPCs) following upper abdominal surgery ranges from 11.4 to 13.1%, increasing to 20–27% after open procedures [1–3]. PPCs are associated with prolonged hospitalization, increased healthcare costs, and higher perioperative mortality rates [4, 5]. Although the ARISCAT integrates preoperative predictors, it does not fully consider perioperative physiological changes [6]. Diaphragmatic dysfunction, a common consequence after upper abdominal surgery, results from multiple perioperative factors, including surgical trauma, incisional pain, anesthesia-related phrenic inhibition, neuromuscular blockade, prolonged immobilization, and systemic inflammation [7–10]. This dysfunction impairs respiratory mechanics and may contribute to the development of PPCs [11–13].

Traditional assessments of diaphragmatic function, such as fluoroscopy and transdiaphragmatic pressure measurements, are limited by radiation exposure or invasiveness [14, 15]. In contrast, bedside diaphragmatic ultrasound offers a non-invasive, radiation-free, and real-time alternative to quantify diaphragmatic dysfunction through validated parameters, including diaphragm thickening fraction (DTF) and diaphragm excursion (DE) during various respiratory maneuvers [16–18]. As an alternative technique, SONOTEPS (ultrasound-guided transcutaneous electrical phrenic nerve stimulation) bypass voluntary effort but require deep sedation [19] and is thus unsuitable for our study’s alert and cooperative postoperative cohort. Importantly, diaphragmatic ultrasound has been validated as a reliable surrogate for inspiratory muscle strength and pulmonary capacity, particularly through DE and DTF measurements [20–22]. While diaphragmatic ultrasound parameters have proven valuable for predicting postoperative outcomes such as difficult weaning and prolonged ICU stays [8, 21], their role in anticipating PPCs remains underexplored, with existing studies reporting inconsistent predictive performance [12, 23, 24].

In this study, perioperative diaphragmatic ultrasound was systematically evaluated in patients undergoing major upper abdominal surgery, to identify the most effective diaphragm ultrasound parameters for predicting PPCs and assess their predictive performance.

## Materials and methods

### Study design

This single-center prospective observational cohort study was conducted at Peking University First Hospital (Beijing, China). The study protocol was approved by the Institutional Ethics Committee of Peking University First Hospital (Approval No. 2018 [249]; January 9, 2019) and registered with the Chinese Clinical Trial Registry (Registration No. ChiCTR1900022859; April 28, 2019). The study was funded by the Youth Clinical Research Project of Peking University First Hospital (Grant No. 2018CR03).

### Participants

Patients were included if they were: (1) aged ≥ 50 years (aligning with ARISCAT [6]); (2) scheduled for major upper abdominal surgery (hepatobiliary and pancreatic head procedures) under general anesthesia, with an anticipated duration of ≥ 2 h and an incision length of ≥ 5 cm; and (3) expected to be extubated within 12 h postoperatively. Patients were excluded if they met any of the following criteria: (1) emergency surgery; (2) American Society of Anesthesiologists (ASA) Physical Status of IV or higher; (3) active pulmonary conditions requiring medical intervention during the past month; (4) neurological disorders diagnosed within the past 3 months that could impair diaphragmatic function; (5) planned phrenic nerve or diaphragmatic surgery; (6) inability to cooperate with diaphragmatic ultrasound assessment; or (7) ​epidural anesthesia use.

### Patient recruitment

Patients were recruited during the preoperative assessment conducted one day before surgery. Eligible individuals who met the inclusion criteria, and provided written informed consent underwent baseline data collection, including demographics, comorbidities, and surgical and anesthetic details. Diaphragmatic ultrasound assessments were performed on preoperative day 1 (PreD1, assessed within 24 h before surgery) and on postoperative day 1 (POD1, assessed within 24 h after surgery).

### Measurement technique

Diaphragmatic ultrasonography was performed using the standardized technique for critically ill patients [16]. Assessments were performed with patients in a semi-recumbent position (head elevated ≥ 30°–45°). Patients were conscious and spontaneously breathing during all diaphragmatic ultrasound assessments. All examinations were conducted using a Philips CX50 color Doppler ultrasound system (Philips China Investment Co. Ltd., Shanghai, China).

To ensure consistency across measurements, standardized respiratory maneuvers were performed as follows: (1) Quiet breathing: normal tidal breathing at rest without verbal prompts; (2) Deep breathing: maximal inspiration through the mouth to total lung capacity, followed by passive exhalation, and (3) Sniff breathing: rapid, forceful nasal inhalations lasting approximately 1 s. Both deep and sniff breathings were performed at 5-second intervals to prevent fatigue.

For diaphragmatic thickening fraction (DTF) assessment, a high-frequency linear-array transducer (L12-3, 3–12 MHz) was positioned at the right anterior axillary line at the ninth intercostal space, aligned parallel to the intercostal space. During deep breathing maneuvers, M-mode imaging was used to measure diaphragmatic thickness at end-inspiration (maximal deep breath) and end-expiration (quiet tidal breathing) over three consecutive respiratory cycles. DTF was assessed only during deep breathing due to unreliable quantification in quiet breathing and technical challenges with sniff breathing [16, 25]. The right diaphragmatic thickening fraction (RD-TF) was calculated using the formula: RD-TF (%) = [(End-inspiratory thickness − End-expiration thickness) / End-expiration thickness] × 100. The average value of the three measurements was recorded.

For diaphragmatic excursion (DE) assessment, a low-frequency curvilinear transducer (C5-1, 1–5 MHz) was positioned below the right costal margin between the midclavicular and anterior axillary lines, angled cranially to ensure that the ultrasound beam was perpendicular to the posterior diaphragm. DE was measured in M-mode as the vertical distance between the lowest (end-expiration) and highest (end-inspiratory) points of the diaphragmatic curve. Measurements were obtained during three respiratory maneuvers: quiet breathing (RQ-DE), deep breathing (RD-DE), and sniff breathing (RS-DE). For quiet breathing, the average of three consecutive measurements was recorded; for deep and sniffing breathing, the highest value from three attempts was recorded to reflect peak diaphragmatic motion.

Postoperative diaphragmatic ultrasound was performed on POD1 using the same equipment and patient positioning as in the preoperative assessment. If the patient’s numerical rating scale (NRS) pain score was ≥ 4 despite patient-controlled analgesia (PCA), rescue intravenous analgesia (flurbiprofen axetil 50 mg as first-line​ or morphine 3 mg for refractory pain) was administered, and the ultrasound assessments were delayed until the pain score decreased to < 4. All assessments were performed by two physicians with more than one year of specialized experience in diaphragmatic ultrasonography, who received standardized training every 3 months and were blinded to the patients’ clinical outcomes. All diaphragmatic parameters were labeled with pre- and post- prefixes to distinguish preoperative and postoperative measurements.

### Surgical and anesthetic management

Before the induction of general anesthesia, the attending anesthesiologist determined the use of regional nerve blocks based on the location and length of the surgical incision. For eligible patients, either a transversus abdominis plane block or a rectus sheath block was performed bilaterally, with each side receiving 20 mL of 0.375% ropivacaine. Prophylactic antibiotics were administered 30 min before the surgical incision. For postoperative nausea and vomiting prophylaxis, either methylprednisolone 40 mg or dexamethasone 5 mg was administered intravenously before induction of anesthesia.

General endotracheal anesthesia was induced using propofol (1.5–2.5 mg/kg) and/or etomidate (0.2–0.3 mg/kg), sufentanil (0.3–0.5 µg/kg), and rocuronium (0.6–1.0 mg/kg). Anesthesia was maintained with target-controlled infusions of propofol (effect-site concentration 1–4 µg/mL) and remifentanil (1–6 ng/mL), along with intermittent boluses of sufentanil (5–20 µg). Neuromuscular blockade was maintained with rocuronium or cisatracurium, as needed. The depth of sedation was monitored using the bispectral index (BIS), with a target range of 40–60. Intraoperative mechanical ventilation was delivered in volume-controlled mode with a tidal volume of 6–8 mL/kg predicted body weight, positive end-expiratory pressure of 3–5 cm H₂O, and a respiratory rate adjusted to maintain end-tidal carbon dioxide between 35 and 40 mmHg.

Following surgery, patients were transferred to the post-anesthesia care unit or intensive care unit (ICU) and extubated as soon as they met the established extubation criteria. Postoperative analgesia was initiated immediately using intravenous PCA. The PCA regimen consisted of sufentanil 150 µg combined with either dexmedetomidine 100 µg or flurbiprofen axetil 200 mg, diluted in 100 mL of normal saline. PCA pump settings included a background infusion rate of 1 mL/h, a bolus dose of 2 mL, and an 8-minute lockout interval. Patients were instructed to self-administer boluses if their NRS pain score reached ≥ 4. PCA was continued for up to 72 h postoperatively, with additional rescue analgesia administered at the discretion of the clinical team.

### Postoperative pulmonary complications

PPCs were diagnosed according to the European Perioperative Clinical Outcome criteria [26], which include respiratory infection, respiratory failure, pleural effusion, atelectasis, pneumothorax, bronchospasm, and aspiration pneumonitis. Only PPCs meeting the diagnostic criteria and classified as Clavien–Dindo grade ≥ II (pharmacological or higher intervention) were included in the analysis [27], and comprehensive definitions are provided in Supplementary Table 1. PPCs were monitored for 14 days postoperatively through daily clinical assessments, with chest radiographs and additional tests performed if clinical indicators suggested potential complications. All PPC diagnoses were independently adjudicated by a physician blinded to diaphragmatic ultrasound results and other clinical data.

### Sample size calculation

The minimum sample size was calculated to test the hypothesis that diaphragmatic ultrasound parameters can predict PPCs with an area under the receiver operating characteristic curve (AUC) > 0.50. Based on a prior randomized controlled trial at our institution, the incidence of PPCs undergoing upper abdominal surgeries (including hepatobiliary and pancreatic head surgeries) was 22.2% [28]. We assumed an expected AUC of 0.65 for the ultrasound parameters. Using PASS software (version 15.0, NCSS PASS, USA), a two-sided test with a significance level of α = 0.05 and 90% power (β = 0.10) indicated that 225 participants would be required. To account for potential dropouts owing to factors such as changes in surgical plans, surgery cancellations, or poor-quality ultrasound images, a dropout rate of 10% was estimated. Accordingly, the final sample size was adjusted to 250 patients.

### Statistical analysis

All statistical analyses were conducted using SPSS 22.0 (IBM Corp., Armonk, NY, USA). Continuous variables were first assessed for normality using the Kolmogorov–Smirnov test. Normally distributed variables were expressed as mean ± standard deviation (SD) and compared using the independent samples *t*-test. Non-normally distributed variables were reported as median (interquartile range, IQR) and compared using the Mann–Whitney *U* test. Categorical variables were reported as frequencies (percentages, %) and compared using Pearson’s chi-square test or Fisher’s exact test, as appropriate.

For multiple comparisons of diaphragmatic parameters, a Bonferroni correction was applied with an adjusted threshold (*P* < 0.0125). Univariable logistic regression was used to assess the association between all preoperative and postoperative diaphragmatic ultrasound parameters and the occurrence of PPCs, with ​odds ratios (ORs) and 95% confidence intervals (CIs) calculated for each parameter. All parameters were further analyzed using receiver operating characteristic (ROC) curves to determine their predictive performance, quantified by the ​area under the curve (AUC) and 95% CI. The optimal cutoff values for significant diaphragmatic parameters were identified by maximizing the Youden index. Sensitivity, specificity, positive predictive value (PPV), and negative predictive value (NPV) were derived from contingency tables.

Univariable logistic regression was used to screen potential clinical risk factors for PPCs (variables with *P* < 0.10 retained). Candidate predictors included these variables, ARISCAT risk components, and literature-derived covariates. A multivariable logistic regression model with the enter method adjusted for confounders while evaluating diaphragmatic ultrasound parameters for their independent predictive value.

Post-hoc comparative analysis evaluated diaphragmatic ultrasound’s predictive capacity relative to the ARISCAT model, with exploratory integration of both approaches. Adjusted odds ratios (ORs) with 95% confidence intervals (CIs) were calculated for each model using multivariable logistic regression with the enter method. Model discrimination was compared via differences in area under the curve (AUC) tested with DeLong’s method, applying Bonferroni correction for multiple comparisons (adjusted *P* < 0.0167).​

## Results

**Fig. 1:**
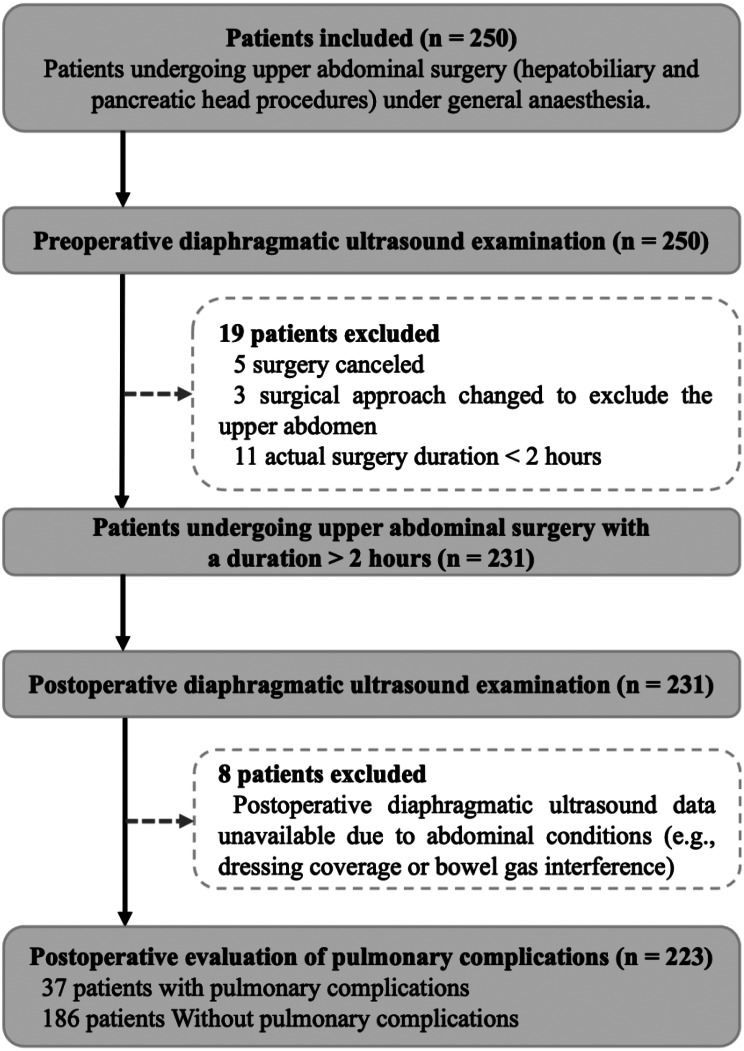
Study Flowchart

Between May 2019 and November 2022, 250 patients were enrolled, and 223 were included in the final analysis (Fig. 1). The mean patient age was 64.9 ± 10.6 years, with 134 (60.1%) patients being male. Overall, 16.6% (37/223) of patients developed PPCs within 14 days postoperatively. The most common PPC was respiratory infection (11.7%, 26/223), followed by atelectasis (9.4%, 21/223), pleural effusion (8.5%, 19/223), acute respiratory failure (5.4%, 12/223), and bronchospasm (2.7%, 6/223). No pneumothorax or aspiration pneumonitis cases were observed. Clinical and perioperative characteristics stratified by PPC occurrence are detailed in Table 1.

**Table 1:** Clinical and perioperative characteristics of patients stratified by postoperative pulmonary complications (PPCs)

	All Patients (*n* = 223)	PPCs (*n* = 37)	Non-PPCs (*n* = 186)	*P* value
Baseline data				
Age, years, mean ± SD	64.9 ± 10.6	68.2 ± 8.6	64.3 ± 10.9	0.042
Male, n (%)	134 (60.1%)	20 (54.1%)	114 (61.3%)	0.412
Body mass index, kg/m^2^, mean ± SD	23.6 ± 3.3	24.3 ± 4.4	23.5 ± 3.0	0.079
**Comorbidities**,** n (%)**				
COPD ^a^	4 (1.8%)	2 (5.4%)	2 (1.1%)	0.129
Asthma ^a^	0 (0.0%)	0 (0.0%)	0 (0.0%)	-
OSAHS	4 (1.8%)	2 (5.4%)	2 (1.1%)	0.129
Coronary artery disease	30 (13.5%)	6 (16.2%)	24 (12.9%)	0.590
Hypertension	91 (40.8%)	14 (37.8%)	77 (41.4%)	0.687
Diabetes, n (%)	68 (30.5%)	13 (35.1%)	55 (29.6%)	0.502
Cerebrovascular disease ^b^, n (%)	16 (7.2%)	5 (13.5%)	11 (5.9%)	0.102
**Smoking status**,** n (%)**				0.322
Never smoked	148 (66.4%)	22 (59.5%)	126 (67.7%)	
Current smoker	29 (13.0%)	4 (10.8%)	25 (13.4%)	
Former smoker ^c^	46 (20.6%)	11 (29.7%)	35 (18.8%)	
**Preoperative examination**				
Anemia (Hb ≤ 10 g/dL), n (%)	13 (5.8%)	3 (8.1%)	10 (5.4%)	0.475
Hypoxemia (SpO₂ ≤ 95%), n (%)	37 (16.6%)	8 (21.6%)	29 (15.6%)	0.368
ASA classification, n (%)				0.014
I-II	148 (66.4%)	17 (45.9%)	131 (70.4%)	
III	75 (33.6%)	20 (54.1%)	55 (29.6%)	
Charlson Comorbidity Index, point, median (IQR)	4.0 (2.0–6.0)	4.0 (2.0–6.0)	4.0 (2.0–6.0)	0.461
**Intraoperative data**				
Anesthesia type, n (%)				0.137
General anesthesia alone	31 (13.9%)	8 (21.6%)	23 (12.4%)	
Combined regional-general anesthesia	192 (86.1%)	29 (78.4%)	163 (87.6%)	
Intraoperative medications				
Norepinephrine, n (%)	16 (7.2%)	3(8.1%)	13(7.0%)	0.734
Rocuronium, mg, median (IQR)	50.0 (50.0–73.0)	50.0 (50.0–50.0)	50.0 (50.0-76.3)	0.460
Fluid balance, ×100 ml, median (IQR)	22.5 (17.0-31.5)	27.0 (21.5–34.0)	22.0 (16.9–31.1)	0.016
Intraoperative blood loss, ml, median (IQR)	200.0 (100.0-460.0)	360.0 (175.0-750.0)	200.0 (100.0-412.5)	0.007
Mechanical ventilation ^d^, h, median (IQR)	5.13 (4.27–7.12)	6.32 (5.15–7.75)	4.92 (4.11–6.68)	< 0.001
Tidal volume, ml, median (IQR)	480.0 (450.0-520.0)	460.0 (445.0-500.0)	480.0 (450.0-520.0)	0.132
PEEP, cm H₂O, median (IQR)	4.0 (3.0–4.0)	4.0 (3.0–4.0)	4.0 (3.0–4.0)	0.152
Anesthesia duration, h	4.97 (4.07–6.23)	5.90 (5.08–6.83)	4.74 (3.95–6.05)	< 0.001
Surgery type, n (%)				0.001
Hepatobiliary surgery	126 (56.5%)	12 (32.4%)	114 (61.3%)	
Pancreatic surgery	97 (43.5%)	25 (67.6%)	72 (38.7%)	
Surgical approach, n (%)				0.006
Laparoscopic	73 (32.7%)	5 (13.5%)	68 (36.6%)	
Open	150 (67.3%)	32 (86.5%)	118 (63.4%)	
Incision length, cm, median (IQR)	20.0 (8.0–29.0)	25.0 (17.5–30.0)	15.0 (6.0–25.0)	< 0.001
Surgery duration, h, median (IQR)	4.17 (3.33–5.43)	4.75 (4.07–6.20)	4.02 (3.23–5.31)	0.001
**Postoperative data**				
NRS of pain-at rest, point, median (IQR)	3.0 (2.0–3.0)	3.0 (2.0–4.0)	2.0 (2.0–3.0)	0.002
NRS of pain-on coughing, point, median (IQR)	4.0 (3.0–5.0)	5.0 (4.0–6.0)	4.0 (3.0–5.0)	0.002
ICU admission, n (%)	55 (24.7%)	18 (48.6%)	37 (19.9%)	< 0.001
Length of hospital stay, d, median (IQR)	20.0 (14.0–31.0)	35.0 (23.5–46.0)	18.5 (14.0-27.3)	< 0.001

Notes:Data are presented as mean ± standard deviation, median (interquartile range), or n (%). Statistical tests:Independent samples *t-*test was used for normally distributed continuous variables (e.g., age, BMI); Mann-Whitney U test for non-normally distributed variables (e.g., incision length, intraoperative blood loss, surgery duration); Pearson’s chi-square test or Fisher’s exact test (for expected cell frequencies < 5) for categorical variables (e.g., COPD, OSAHS). ^a^ COPD and asthma are diagnosed by a pulmonologist; ^b^ Diagnosed by a neurologist; ^c^ Former smoker is defined as cessation of smoking for ≥ 4 weeks; ^d^ Mechanical ventilation time includes both intraoperative and postoperative durations. Abbreviations:PPCs, postoperative pulmonary complications; BMI, body mass index; COPD, chronic obstructive pulmonary disease; OSAHS, obstructive sleep apnea-hypopnea syndrome; ASA, American Society of Anesthesiologists physical status classification; PEEP, positive end-expiratory pressure; NRS, Numerical Rating Scale (0–10, 0 = no pain, 10 = worst pain); ICU, intensive care unit

**Fig. 2:**
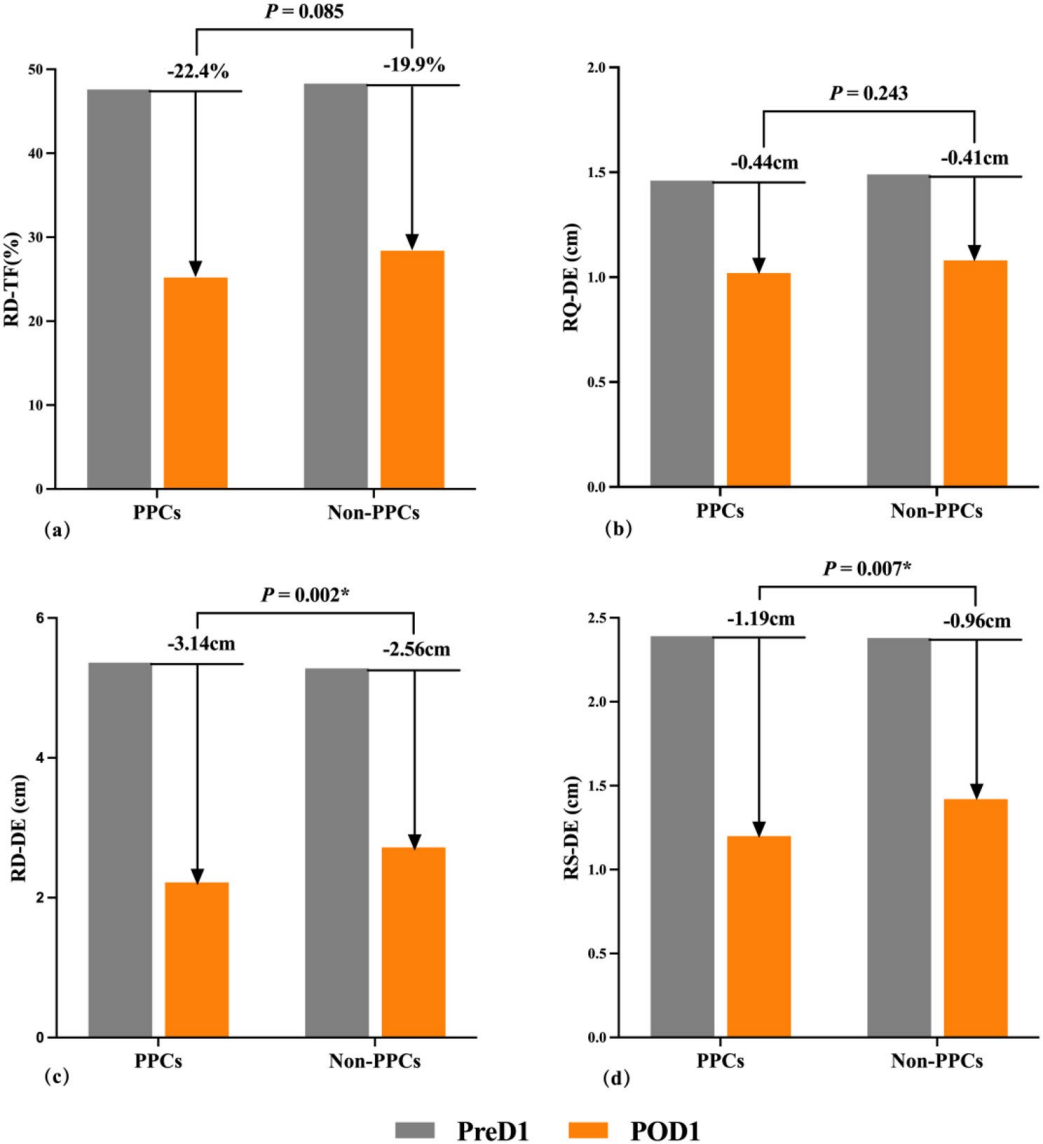
Comparison of diaphragmatic parameters between patients with and without postoperative pulmonary complications (PPCs) Total cohort: N = 223 (PPCs group: n = 37; non-PPCs group: n = 186). All diaphragmatic parameters demonstrated significant postoperative reductions on postoperative day 1(POD1) compared to preoperative day 1 (PreD1) (*P* < 0.001, paired t-test). Specifically, (**a**) Patients with PPCs exhibited a numerically greater postoperative reduction in diaphragmatic thickening fraction (RD-TF) compared to those without PPCs (ΔRD-TF = −22.4% vs. −19.9%, *P* = 0.085); (**b**) A similar trend was observed for diaphragmatic excursion during quiet breathing (RQ-DE), with PPCs showing a numerically greater reduction (ΔRQ-DE = −0.44 cm vs. −0.41 cm, *P* = 0.243); (**c**) Diaphragmatic excursion during deep breathing (RD-DE) showed a significantly greater reduction in patients with PPCs (ΔRD-DE = −3.14 cm vs. −2.56 cm, *P* = 0.002); (**d**) Similarly, diaphragmatic excursion during sniff breathing (RS-DE) decreased significantly more in the PPC group (ΔRS-DE = −1.19 cm vs. −0.96 cm, *P* = 0.007). * Bonferroni correction for multiple comparisons (adjusted *P* < 0.0125) confirmed significantly greater reduction in the PPCs group for RD-DE and RS-DE. Abbreviations:PPCs, postoperative pulmonary complications; RD-TF: right diaphragmatic thickening fraction during deep breathing (%); RQ-DE: right diaphragmatic excursion during quiet breathing (cm); RD-DE: right diaphragmatic excursion during deep breathing (cm); RS-DE: right diaphragmatic excursion during sniff breathing (cm); PreD1: preoperative day 1; POD1: postoperative day 1

In the full cohort, diaphragmatic ultrasound parameters on POD1 were significantly reduced when compared to preoperative values (*P* < 0.001). Among these, RD-DE decreased by 50.0% and RS-DE decreased by 41.8% from preoperative baselines, with significantly greater reductions observed in patients who developed PPCs compared to those without PPCs (ΔRD-DE: −3.14 cm vs. −2.56 cm, *P* = 0.002; ΔRS-DE: −1.19 cm vs. −0.96 cm, *P* = 0.007) (Fig. 2). Univariable analyses demonstrated that higher post-RD-DE (OR = 0.500, 95% CI: 0.322–0.777; *P* = 0.002) and post-RS-DE (OR = 0.296, 95% CI: 0.123–0.716; *P* = 0.007) were each inversely associated with a decreased risk of PPCs (Table 2). ROC analysis demonstrated AUC values of 0.669 (95% CI: 0.576–0.762; *P* = 0.001) for post-RD-DE and 0.636 (95% CI: 0.538–0.734; *P* = 0.009) for post-RS-DE; both parameters reached statistical significance under Bonferroni-adjusted significance threshold (*P* < 0.0125) (Table 2). Their complete diagnostic performance metrics (sensitivity, specificity, PPV, NPV) are provided in Table 3​​. The composite index post-RDS-DE, derived by summing the two independent predictors (post-RD-DE + post-RS-DE), demonstrated improved predictive ability for PPCs, with an AUC of 0.680 (95% CI: 0.587–0.773; *P* = 0.001) (Table 2). Collinearity diagnostics revealed acceptable variance inflation factors (VIF = 1.166, tolerance = 0.858) between RD-DE and RS-DE (Pearson’s *r* = 0.377, *P* < 0.001), indicating their complementary contributions.

**Table 2:** Comparison of preoperative and postoperative diaphragmatic ultrasound parameters

	All Patients (*n* = 223)	PPCs (*n* = 37)	Non-PPCs (*n* = 186)	OR (95% CI)	*P* value ^†^	AUC (95% CI)	*P* value ^‡^
Preoperative, mean ± SD							
pre-RD-TF, %	48.2 ± 12.7	47.6 ± 13.3	48.3 ± 12.6	0.638 (0.039–10.32)	0.752	0.508 (0.407–0.609)	0.052
pre-RQ-DE, cm	1.48 ± 0.34	1.46 ± 0.29	1.49 ± 0.35	0.765 (0.264–2.223)	0.623	0.528 (0.435–0.620)	0.047
pre-RD-DE, cm	5.28 ± 1.15	5.36 ± 0.88	5.28 ± 1.20	1.062 (0.784–1.439)	0.699	0.444 (0.348–0.541)	0.049
pre-RS-DE, cm	2.39 ± 0.64	2.39 ± 0.56	2.38 ± 0.66	1.030(0.597–1.778)	0.915	0.465 (0.370–0.561)	0.049
Postoperative, mean ± SD							
post-RD-TF, %	27.8 ± 10.2	25.2 ± 9.9	28.4 ± 10.2	0.037 (0.001–1.579)	0.085	0.615 (0.516–0.714)	0.027
post-RQ-DE, cm	1.07 ± 0.32	1.02 ± 0.32	1.08 ± 0.32	0.511 (0.165–1.579)	0.243	0.578 (0.478–0.679)	0.133
post-RD-DE, cm	2.64 ± 0.90	2.22 ± 0.80	2.72 ± 0.89	0.500 (0.322–0.777)	0.002	0.669 (0.576–0.762)	0.001
post-RS-DE, cm	1.39 ± 0.45	1.20 ± 0.42	1.42 ± 0.44	0.296 (0.123–0.716)	0.007	0.636 (0.538–0.734)	0.009
post-RDS-DE, cm	4.03 ± 1.14	3.42 ± 1.06	4.15 ± 1.12	0.549 (0.391–0.772)	< 0.001	0.680 (0.587–0.773)	< 0.001

Notes:Data are presented as mean ± standard deviation, median (interquartile range), or n (%). Statistical tests:^†^ Binary logistic univariate analysis: *P*-values evaluated against a Bonferroni-adjusted threshold (*P* < 0.0125) for 4 comparisons. ^‡^ Receiver operating characteristic (ROC) analysis: Bonferroni-corrected threshold (*P* < 0.0125) applied to AUC comparisons. Abbreviations:PPCs, postoperative pulmonary complications; RD-TF: right diaphragmatic thickening fraction during deep breathing; RQ-DE: right diaphragm excursion during quiet breathing; RD-DE: right diaphragm excursion during deep breathing; RS-DE: right diaphragm excursion during sniff breath; RDS-DE: a composite diaphragm index calculated as the sum of RD-DE and RS-DE; pre-: Preoperative day 1; post-: Postoperative day 1

**Table 3:** Diagnostic performance of postoperative diaphragmatic ultrasound indices for predicting postoperative pulmonary complications (PPCs)​​

	Cutoff Value ^†^	Sensitivity (%)	Specificity (%)	PPV (%)	NPV (%)
post-RD-DE	< 2.18 cm	64.9	72.6	32.0	91.2
post-RS-DE	< 1.54 cm	86.5	37.1	21.5	93.2
post-RDS-DE	< 3.55 cm	62.2	72.6	31.1	90.6

Statistical tests:

^†^ Cutoff determination by maximizing Youden’s index; diagnostic metrics derived from contingency tables

Abbreviations:

PPCs, postoperative pulmonary complications; RD-DE: right diaphragm excursion during deep breathing; RS-DE: right diaphragm excursion during sniff breath; RDS-DE: a composite diaphragm index calculated as the sum of RD-DE and RS-DE; post-: Postoperative day 1

**Fig. 3:**
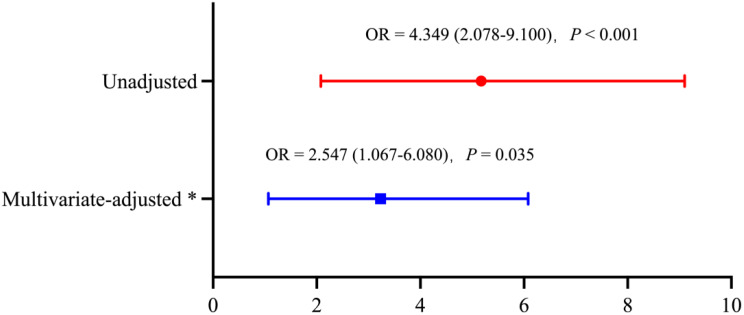
Predictive ability of post-RDS-DE < 3.55 cm for the occurrence of PPCs in patients undergoing upper abdominal surgery *The adjusted odds ratio for post-RDS-DE < 3.55 cm was derived from a multivariable logistic regression model that adjusted for 12 covariates. Adjustment variables were selected through a three-stage process: (a) Univariate screening (*P* < 0.10): ASA class III, incision length (cm), intraoperative blood loss (mL), postoperative NRS (coughing) score, and post-RDS-DE < 3.55 cm; (b) ARISCAT risk components: Age (>80 yr), preoperative anemia (Hb ≤ 10 g/dL), preoperative hypoxemia (SpO₂ ≤ 95%), and duration of surgery (>3h), preserving alignment with the ARISCAT risk model framework; (c) Literature-derived covariates: BMI (kg/m2), chronic respiratory disease (COPD, asthma, or OSAHS), and laparoscopic approach, based on established clinical relevance. Post-RDS-DE <3.55 cm remained an independent predictor of PPCs (adjusted OR = 2.547, 95% CI: 1.067–6.080; *P* = 0.035) after adjustment. Abbreviations:RDS-DE: a composite diaphragm index calculated as the sum of RD-DE and RS-DE; ASA: American Society of Anesthesiologists Physical Status Classification; ARISCAT: Assess Respiratory Risk in Surgical Patients in Catalonia; BMI, Body Mass Index; COPD, Chronic Obstructive Pulmonary Disease; OSAHS, Obstructive Sleep Apnea-Hypopnea Syndrome

**Fig. 4:**
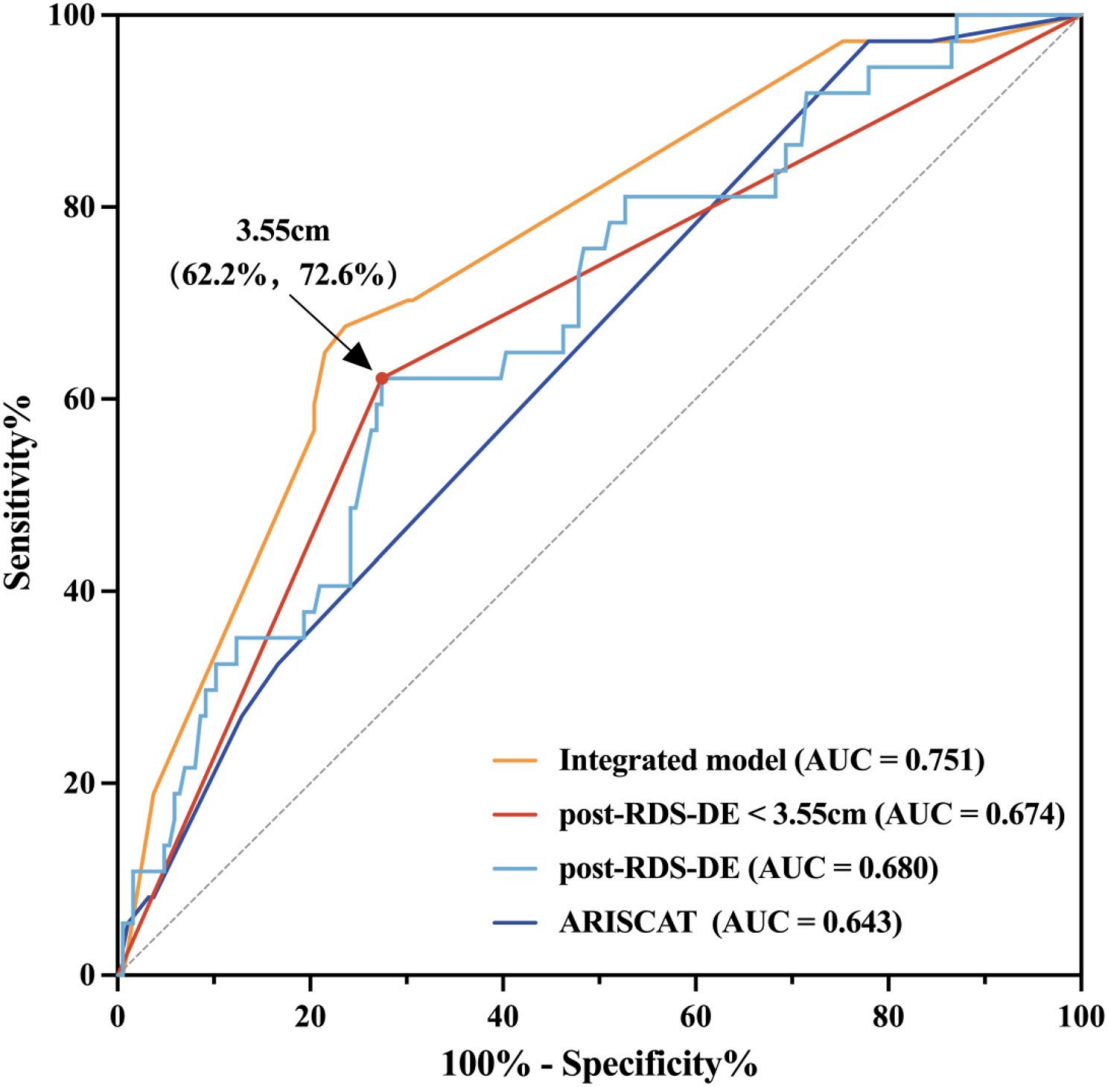
Receiver Operating Characteristic (ROC) Curves for Predicting Postoperative Pulmonary Complications (PPCs) The composite diaphragm index (post-RDS-DE) demonstrated discriminative capacity (AUC = 0.680; 95% CI: 0.587–0.773; *P* = 0.001), with 3.55 cm as the optimal cutoff (sensitivity 62.2%, specificity 72.6%). This defined the dichotomous predictor: post-RDS-DE < 3.55 cm. ROC curves compare the predictive performance of three models: (a) ARISCAT alone: AUC = 0.643 (95% CI: 0.552–0.735); (b) Diaphragmatic ultrasound index alone (post-RDS-DE < 3.55 cm): AUC = 0.674 (95% CI: 0.575–0.772); (c) Integrated model: AUC = 0.751 (95% CI: 0.667–0.836). DeLong’s test with Bonferroni-adjusted significance threshold (*P* < 0.0167): Integrated vs. ARISCAT: *P* = 0.004; Integrated vs. Diaphragmatic ultrasound index: *P* = 0.002; Diaphragmatic ultrasound index vs. ARISCAT: *P* = 0.572. Abbreviations:RDS-DE: a composite diaphragm index calculated as the sum of RD-DE and RS-DE; post-: postoperative day 1; ARISCAT: Assess Respiratory Risk in Surgical Patients in Catalonia

The optimal cutoff value for post-RDS-DE was 3.55 cm, yielding a sensitivity of 62.2% and specificity of 72.6%, with a PPV of 31.1% and an NPV of 90.6% (Table 3). Patients with post-RDS-DE < 3.55 cm had a significantly higher incidence of PPCs compared to those with post-RDS-DE ≥ 3.55 cm (31.1% [23/74] vs. 9.4% [14/149], *P* < 0.001). After adjustment for 12 covariates selected via three-stage screening (univariate *P* < 0.10 candidates, ARISCAT risk components, literature-supported predictors), reduced diaphragmatic function (post-RDS-DE < 3.55 cm) remained independently associated with an increased risk of PPCs (adjusted OR = 2.547, 95% CI: 1.067–6.080; *P* = 0.035) (Fig. 3). Building on this predictive framework, post-RDS-DE < 3.55 cm was evaluated as a representative diaphragmatic ultrasound index alongside the established ARISCAT for predicting PPCs.​​ When compared with ​ARISCAT​ (AUC = 0.643, 95% CI: 0.552–0.735), ​ diaphragmatic ultrasound index (post-RDS-DE < 3.55 cm)​ showed a comparable discrimination (AUC = 0.674, 95% CI: 0.575–0.772; DeLong’s *P* = 0.572 vs. ARISCAT). The integration of both predictors achieved a significantly higher AUC of 0.751 (95% CI: 0.667–0.836), with the improvement over ARISCAT alone (DeLong’s *P* = 0.004) surpassing the prespecified Bonferroni-adjusted significance threshold (*P* < 0.0167) (Table 4; Fig. 4).

**Table 4:** Multivariate analysis of predictors in three models for postoperative pulmonary complications (PPCs)​​

	Multivariate Analysis OR (95% CI) ^†^, *N* = 223		
Predictors​	ARISCAT Model ^a^	Diaphragmatic ultrasound Model ^b^	Integrated Model​ ^c^
Aged > 80 yr	1.917 (0.436–8.430)		2.526 (0.515–12.386)
Preoperative hypoxemia (SpO₂ ≤95%)	1.726 (0.693–4.297)		1.503 (0.575–3.924)
Preoperative anemia (Hb ≤ 10 g/dL)	1.056 (0.257–4.346)		0.681 (0.141-3.300)
Duration of surgery > 3 h	10.789 (1.422–81.861)		10.313 (1.339–79.451)
post-RDS-DE < 3.55 cm		4.349 (2.078-9.100)	4.343 (2.008–9.393)

Notes:^a^ In the ARISCAT model, four predictors were included while three were excluded: surgical incision site (all patients were upper abdominal) and emergency status (all elective procedures) demonstrated no variability; recent respiratory infection (*n* = 3) had insufficient sample size for reliable estimation; ^b^ In the diaphragmatic ultrasound model, the dichotomous predictor was defined as post-RDS-DE < 3.55 cm; ^c^ In the integrated model, predictors from both ARISCAT model (Age > 80 year, preoperative anemia, preoperative hypoxemia, and duration of surgery > 3 h) and diaphragmatic ultrasound model (post-RDS-DE < 3.55 cm) were combined. Statistical tests:^†^ Adjusted odds ratios (ORs) and 95% confidence intervals (CIs) were derived from multivariable binary logistic regression models using the forced-entry (enter) method. Abbreviations:RDS-DE: composite diaphragm index calculated as the sum of RD-DE and RS-DE; post-: Postoperative day 1; ARISCAT: Assess Respiratory Risk in Surgical Patients in Catalonia

## Discussion

In this prospective observational cohort study, postoperative diaphragmatic dysfunction quantified by the composite index post-RDS-DE was established as an independent predictor of PPCs in older intermediate-high risk patients following upper abdominal surgery (ARISCAT ≥ 26) [6]. The index demonstrated a moderate predictive ability for PPCs (AUC = 0.680), with the optimal cutoff of 3.55 cm achieving a high NPV (90.6%) that offers clinical utility for early identification of lower-risk patients. Multivariate analysis confirmed ​post-RDS-DE < 3.55 cm​ as an independent predictor (adjusted OR = 2.547, *P* = 0.035). Post-RDS-DE alone showed a discriminative ability comparable to ARISCAT (*P* = 0.572); However, their integration significantly outperformed ARISCAT alone (AUC = 0.751 vs. 0.643, *P* = 0.004).

In patients after upper abdominal surgery, various subtypes of PPCs share interconnected pathophysiological pathways rooted in impaired respiratory mechanics, and often coexisting and mutually exacerbating one another [6]. Diaphragmatic dysfunction may play an important role in these interactions by diminishing inspiratory reserve and impairing cough efficacy, and thus potentially increasing the risks of inflammation and atelectasis [10, 11]. Although a few studies explored the role of diaphragmatic ultrasound parameters in predicting PPCs, the existing evidence mainly focused on isolated parameters under single respiratory maneuvers (e.g., deep breathing) [12, 22]. We incorporated sniff breathing, a maneuver validated for evaluating phasic diaphragm activation that correlates with maximal inspiratory pressure (MIP) [29, 30]. By comparing multiple diaphragmatic parameters, we found that reduced DE during deep breathing (AUC = 0.669) and sniff breathing (AUC = 0.636) on POD1 ​both predicted PPCs. Specifically, these parameters reflect complementary functions:​​ baseline ventilatory reserve (RD-DE, sustained contraction during deep breathing) and dynamic-responsive capacity (RS-DE, rapid phasic activation during sniff breathing). Their synergism was statistically confirmed by low collinearity (VIF = 1.166) and moderate correlation (*r* = 0.377). Consequently,​​ we combined the two parameters into a composite index (post-RDS-DE), which enhanced prognostic accuracy (AUC = 0.680) by integrating these distinct physiological mechanisms during maximal inspiratory maneuvers. This dual-assessment approach not only underscores the multifactorial nature of diaphragmatic impairment in the pathogenesis of PPCs but also provides a mechanistic rationale for applying diaphragmatic ultrasound assessment in PPC risk prediction.

Our results showed that a composite index post-RDS-DE of < 3.55 cm provided clinically actionable risk stratification with a high negative predictive value (90.6%), i.e., it effectively excludes patients at lower-risk risk of PPCs. This reduces unnecessary monitoring and optimizes resource allocation. The feasibility of diaphragmatic ultrasonography is supported by the rapid bedside nature of assessment (achievable in 5 min when performed by experienced operators on cooperative patients), which permits direct integration into routine postoperative care protocols. ​However, the moderate sensitivity and specificity of post-RDS-DE, along with its low positive predictive value, underscore the necessity of combining it with established clinical risk models. In this context, the ARISCAT demonstrated inherent limitations as three of its seven predictors (age, surgical site, and emergency status) were homogenized by our inclusion criteria, resulting in suboptimal discriminative performance (AUC = 0.643). While post-RDS-DE alone showed comparable prediction to ARISCAT (AUC = 0.674 vs. 0.643; *P* = 0.572), their multivariate integration achieved ​a statistically significant prognostic improvement​ (AUC = 0.751 vs. ARISCAT alone; ΔAUC = + 0.108, *P* = 0.004). This synergy likely reflects complementary risk stratification: ARISCAT captures baseline clinical vulnerability (e.g., age, comorbidities), while post-RDS-DE quantifies perioperative physiological insults (e.g., diaphragm inhibition induced by anesthesia and surgical trauma). Although our findings remain preliminary due to the limited sample size in the combined model analysis, they position diaphragmatic ultrasound assessment as a promising adjunctive tool.

This study has several limitations. First, we focused on hepatobiliary/pancreatic head surgeries (anatomically adjacent to the right diaphragm) and excluded the left diaphragm (owing to technical challenges in left diaphragmatic imaging). This limits the generalizability of our findings to other surgical populations. Second, we excluded patients receiving epidural analgesia to minimized anesthetic heterogeneity. Our institutional preferred regional nerve blocks over epidurals because of their advantages in reducing hypotension and providing adequate analgesia without motor blockade [31]. This limits the generalizability of our findings to other settings where epidural analgesia remains a standard technique. Third, the lower-than-expected PPC incidence (16.6%), potentially due to inclusion of non-cancer patients (21.2%, 47/223) undergoing shorter surgeries, might underestimate the predictive power of diaphragmatic parameters. Fourth, morphine was administered as rescue analgesia in only 1.3% (3/223) of patients. Although no respiratory compromise was observed, the potential for transient opioid effects on diaphragmic function cannot be entirely excluded. Finally, the single-center design and the sample size (*n* = 223) also limit the generalizability of our results. Further validation through larger, multicenter studies is warranted.

## Conclusion

This study proposes diaphragmatic ultrasonography as a novel functional assessment tool for PPC risk stratification after upper abdominal surgery. By quantifying diaphragmatic dysfunction during maximal inspiratory maneuvers, the composite post-RDS-DE index (cutoff < 3.55 cm) provides high negative predictive value (90.6%), supporting its potential role in identifying patients at lower-risk of PPCs who may require less intensive monitoring. In patients with dysfunction (post-RDS-DE < 3.55 cm), integrating real-time assessment with preoperative ARISCAT risk index improves identification of higher-risk patients by combining dynamic respiratory function evaluation with established risk factors to guide targeted interventions.

Future research should expand validation of diaphragmatic ultrasound in larger cohorts to assess perioperative respiratory impairment and predict PPCs, strategically integrate this approach with existing prediction models to demonstrate synergistic value and implement diaphragmatic ultrasound into clinical practice to guide precise risk stratification and improve current management strategies.

## Supplementary Information

Below is the link to the electronic supplementary material.

Supplementary Material 1

## Data Availability

The datasets supporting the conclusions of this article are included within the article and its additional files.

## Abbreviations

PPCs: Postoperative pulmonary complications
ARISCAT: Assess Respiratory Risk in Surgical Patients in Catalonia
DE: Diaphragmatic excursion
DTF: Diaphragmatic thickening fraction
RD-DE: Right diaphragmatic excursion during deep breathing
RS-DE: Right diaphragmatic excursion during sniff breathing
RQ-DE: Right diaphragmatic excursion during quiet breathing
RDS-DE: a Composite diaphragmatic index (sum of RD-DE and RS-DE)
PreD1: Preoperative day 1
POD1: Postoperative day 1
pre-: Preoperative (prefix)
post-: Postoperative (prefix)
AUC: Area under the receiver operating characteristic curve
NPV: Negative predictive value
SD: Standard deviation
IQR: Interquartile range
ASA: American Society of Anesthesiologists Physical Status Classification
BIS: Bispectral Index
PCA: Patient-controlled analgesia
NRS: Numerical rating scale
ICU: Intensive care unit
MIP: Maximal inspiratory pressure
VIF: Variance inflation factor​
OR: Odds ratio​
